# Zooming in on protein–RNA interactions: a multi-level workflow to identify interaction partners

**DOI:** 10.1042/BST20191059

**Published:** 2020-08-21

**Authors:** Alessio Colantoni, Jakob Rupert, Andrea Vandelli, Gian Gaetano Tartaglia, Elsa Zacco

**Affiliations:** 1 Center for Life Nanoscience, Istituto Italiano di Tecnologia, Viale Regina Elena 291, 00161 Rome, Italy; 2 Department of Biology ‘Charles Darwin’, Sapienza University of Rome, P.le A. Moro 5, Rome 00185, Italy; 3 Center for Human Technologies, Istituto Italiano di Tecnologia, Via Erico Melen 83, 16152 Genoa, Italy; 4 Centre for Genomic Regulation (CRG), The Barcelona Institute for Science and Technology, Dr. Aiguader 88, 08003 Barcelona, Spain; 5 Universitat Pompeu Fabra (UPF), 08003 Barcelona, Spain; 6 Systems Biology of Infection Lab, Department of Biochemistry and Molecular Biology, Biosciences Faculty, Universitat Autònoma de Barcelona, 08193 Cerdanyola del Vallès, Spain; 7 Institucio Catalana de Recerca i Estudis Avançats (ICREA), 23 Passeig Lluis Companys, 08010 Barcelona, Spain

**Keywords:** clip, molecular modelling, protein–RNA interaction predictions, protein–RNA interaction validation, protein–RNA interactions

## Abstract

Interactions between proteins and RNA are at the base of numerous cellular regulatory and functional phenomena. The investigation of the biological relevance of non-coding RNAs has led to the identification of numerous novel RNA-binding proteins (RBPs). However, defining the RNA sequences and structures that are selectively recognised by an RBP remains challenging, since these interactions can be transient and highly dynamic, and may be mediated by unstructured regions in the protein, as in the case of many non-canonical RBPs. Numerous experimental and computational methodologies have been developed to predict, identify and verify the binding between a given RBP and potential RNA partners, but navigating across the vast ocean of data can be frustrating and misleading. In this mini-review, we propose a workflow for the identification of the RNA binding partners of putative, newly identified RBPs. The large pool of potential binders selected by in-cell experiments can be enriched by *in silico* tools such as *cat*RAPID, which is able to predict the RNA sequences more likely to interact with specific RBP regions with high accuracy. The RNA candidates with the highest potential can then be analysed *in vitro* to determine the binding strength and to precisely identify the binding sites. The results thus obtained can furthermore validate the computational predictions, offering an all-round solution to the issue of finding the most likely RNA binding partners for a newly identified potential RBP.

## Introduction

Since their discovery and until recently, RNA-binding proteins (RBPs) have been identified by the presence of one or more RNA-binding domains in their sequences [1]. However, concomitantly to a new appreciation for RNA as key biological macromolecule acting at post-transcriptional level [2–4], there has also been a re-evaluation of what constitutes an RBP. Since one of the principal ways by which RNA exerts its function is by the formation of ribonucleoprotein complexes, every protein capable of establishing even weak and extemporary interactions with an RNA molecule may be defined as RBP [5,6]. The interactions of proteins with RNA can be highly dynamic and heavily dependent on the cellular environment [7], which makes the goal of defining the range of affinities and specificities quite challenging [8]. In fact, indiscriminate binding of RNA by RBPs is a quite common phenomenon [9], and the assumption that stronger affinity translates into more relevant biological functions is not necessarily correct [10]. For the scientific community, the revelation of the dynamicity and malleability of the partnership between RBPs and RNA allows for the exploration of new possible interaction mechanisms, networks, genes and protein regulation systems to investigate. It becomes, therefore, increasingly important to complete the catalogue of eukaryotic RBPs, at present containing >315 000 elements (about 6000 orthologues from >150 species) of which 3500 are from *H. sapiens* and can be divided in conventionally-defined RBPs and several classes of non-canonical RBPs [5,11,12].

Large-scale identification of new potential RBPs can reveal unexpected biological and pathological functions and, when confronted with a novel RBP, the identification of its RNA binding partners is a critical step to define the protein's cellular and molecular roles. To achieve this goal, increasingly sophisticated high-throughput methodologies have been developed, spanning from methods that aim to preserve the native cellular RNA–RBP interactions [13–15] to finely-controlled *in vitro* techniques that allow to define kinetics and dynamic parameters for each binding pair [16,17]. However, the field that has probably seen the biggest evolution in the shortest time span is the one of computational prediction algorithms [18–22]. The wide range of computational tools available includes several data-driven methods based on learning models, in which the algorithms are trained using experimental outcomes and databases to identify RNA–RBP binding patterns and define the genome-wide profiling of RNA–protein interactions [22].

In this short review, we propose a work pipeline for the identification of the RNA binding partners for novel putative RBPs. Starting from in-cell data harvesting, we would like to guide the reader through the employment of the different tools offered by *cat*RAPID [23,24], our in-house developed RNA–protein interaction prediction algorithm, and propose some indications on how to validate the outcome experimentally. Our wider goal is to support the scientific community in the identification of novel biologically relevant non-canonical RBPs.

## Identification of RNA binding partners in cellular context

**Figure 1.**
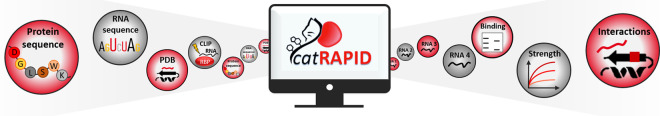
The workflow of discovering RNA partners for an RBP. Protein and RNA sequence databases, structural information and results from RIP/CLIP experiments feed computational prediction tools such as *cat*RAPID. The software utilises this information to define RNA sequences with high probability of interacting with a given RBP and rank them accordingly. Several *in vitro* techniques allow for the validation of predicted results, for the calculation of binding strength and the definition of the binding sites.

The validation and training of computational algorithms for the prediction of protein-RNA interactions is strongly supported by experimental data (Figure 1). Prediction software can be significantly enriched by the output of techniques able to identify a protein's RNA partners within the cellular environment; such procedures are key to defining native interaction pairs and to monitoring responses and variations upon physiological *stimuli* or under pathological stress.

### RIP-based approaches

The main tool to obtain information about the RNA binding partners of a target protein in the cellular environment is immunoprecipitation (IP), a widespread technique to pull down the protein of interest together with its physiological RNA binding partners. RNA immunoprecipitation (RIP) requires incubation of cell lysates with an antibody raised against the target protein [25]. RNA molecules bound to the target protein can then be isolated and analysed to reconstruct physiological native complexes formed within the cell. RIP can be coupled to either microarrays (RIP-Chip) or high-throughput sequencing (RIP-Seq). In either case, RIP can only determine the identity of the RNA molecules associated to the target protein, unless digestion-optimized RIP (DO-RIP) is performed [26]. This variation of RIP introduces an RNase digestion step to preserve only the portion of RNA bound to the protein, allowing for binding-site mapping [27]. If, instead, the interest is focused on identifying multi-subunit ribonucleoproteins, the most appropriate RIP variant may be RIP in tandem (RIPiT), which employs two distinct IP steps performed either with antibodies against different proteins of the complex or with antibodies binding different regions of the same target protein [28]. Information about native protein–RNA complexes formed within the cell can also be obtained by employing affinity tags [29], without having to rely on the antibody's specificity and sensitivity.

### CLIP-based approaches

To overcome RIP's limitations (enrichment of indirectly bound RNAs, detection of interactions not present in cell but formed after lysis, loss of weaker interactions due to the required stringent washing conditions), cross-linked RNA immunoprecipitation (CLIP) has been developed [30]. CLIP promotes the stabilisation of the bonds between a protein and its interacting RNA, generally by UV radiation [31]. A large amount of CLIP variants is available, and most of them can offer high-resolution results at the single nucleotide level. Pioneers among these are, for example, the high-throughput sequencing CLIP (HITS-CLIP), that enriches the RNA population for sequences corresponding to the RBP binding sites [32]; the individual nucleotide-resolution CLIP (iCLIP) [33,34] and enhanced CLIP (eCLIP) [35], that utilise different oligonucleotide adapter configurations to obtain RNAs of different lengths employed to build the interacting fragments at single-nucleotide resolution; and the photoactivatable ribonucleoside CLIP (PAR-CLIP), that relies on metabolic incorporation of labelled ribonucleoside analogues that yield photo-adducts when cross-linked at selected wavelengths [36]. Radiation-free CLIP variants, such as infrared-CLIP (irCLIP), have also been reported [37]. To overcome the restrictions imposed by the use of irreversible UV cross-linking, formaldehyde-based techniques such as fCLIP, which are more efficient in capturing interactions with dsRNA [38], can also be employed [39]. One limit of CLIP-Seq approaches is that low crosslinking efficiency makes low abundant transcripts difficult to detect, while transcripts present at high amounts are usually over-represented in IP samples. This issue can be partial solved with a proper bioinformatic analysis.

### Bioinformatic analysis

**Figure 2.**
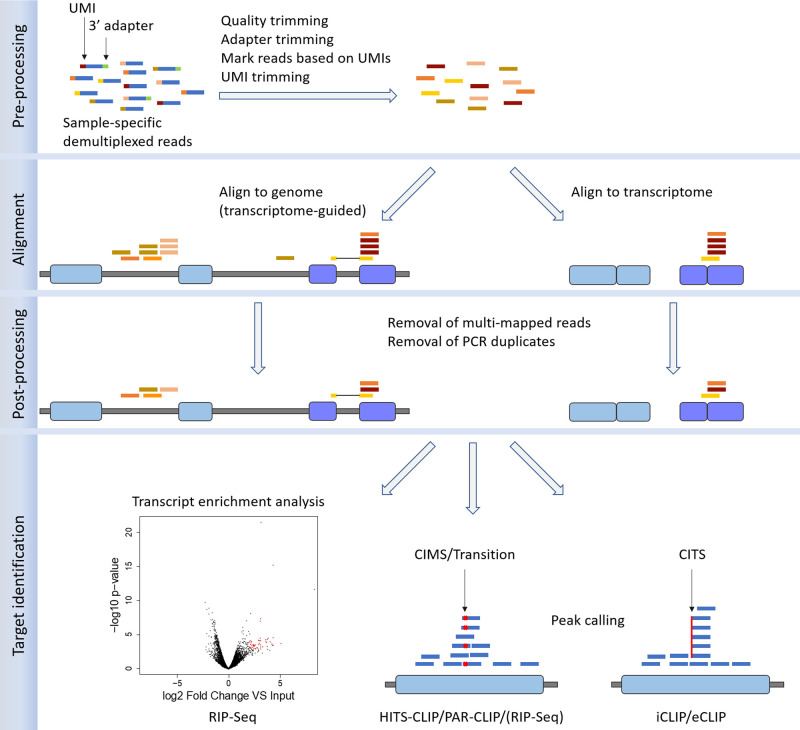
Bioinformatics analysis of CLIP-Seq and RIP-Seq data. After being de-multiplexed based on sample-specific barcodes, reads undergo a **pre-processing** phase. UMIs are not always used, being more common in iCLIP and eCLIP protocols. When employed, they are sometimes used to remove PCR duplicates directly at this stage, but in most cases reads are simply marked based on UMI sequence, as shown by the colours assigned to trimmed reads. After the **alignment** of reads to the genome or to the transcriptome is performed, **post-processing** is needed to filter out multi-mapped reads and to collapse reads mapping at the same position, that are likely to represent PCR duplicates; if UMI-based read marking occurred, natural duplicates, which map at the same place but have different UMIs, can be retained, as shown here. **RNA target identification** and binding site detection strategy depend on the protocol. Roughly, such approaches can be divided into transcript enrichment analysis (RIP-Seq), which is analogous to differential expression analysis, and peak-calling (all protocols). Single-nucleotide resolution can be achieved using CIMSs in HITS-CLIP, transitions in PAR-CLIP and CITSs in iCLIP/eCLIP. HITS-CLIP experiments do not always produce clear and usable CIMS patterns.

High-throughput sequencing of the RNAs isolated through RIP, CLIP and related protocols yields millions of short ‘reads’, which represent the sequenced portions of cDNA fragments obtained through RNase titration (a step omitted in standard RIP-Seq), followed by reverse transcription and PCR amplification. The RNase treatment allows to obtain fragments long enough to be uniquely mappable but short enough to identify the binding site with the highest possible resolution. Library preparation ends with the production of cDNA fragments flanked by adapters that allow amplification and sequencing, resulting in the generation of short reads that can undergo bioinformatic analysis aimed at identifying RNA targets for the RBP (Figure 2). Reads pre-processing steps, including demultiplexing and adapter trimming, are often required, especially in those cases in which Unique Molecule Identifiers (UMIs) are used [40]. UMIs are random barcodes which identify unique cDNA fragments, allowing to detect and remove PCR duplicates that are commonly produced during CLIP-Seq library preparation [34,35].

To identify the RNA molecules from which they derive, reads are aligned to a reference genome using splice-aware alignment programs commonly used to analyze standard RNA-Seq data, like TopHat2 [41] or Star [42]. If the RBP under investigation binds mature mRNAs, reads can be mapped directly to the transcriptome [43] using a splice-unaware mapper like Bowtie2 [44]. Reads coming from HITS-CLIP experiments have high mutation rates (usually deletions) at the cross-linking site (CIMS, standing for cross-linking induced mutation sites), which are due to residual amino acids hindering the reverse transcriptase [45–47]. Similarly, the use of 4-thiouridine (4-SU) or 6-thioguanosine (6-SG) in the PAR-CLIP protocol leads to a high number of transition events (T to C or G to A, respectively) at the cross-linking sites [48]. Reads mapping can be improved by taking into account the high rate of such mutations. For instance, the splice-unaware BWA aligner [49] has been modified in order to incorporate an error model that favours PAR-CLIP specific transitions [50,51]. Reads produced by iCLIP, and likely eCLIP, experiments do not require such special treatment, since such protocols enrich for cDNAs truncated at the cross-linking site (CITS, standing for cross-linking induced truncation sites), while only a minor proportion of fragments represent CIMS-containing read-through cDNAs [43].

Post-processing of aligned reads is a mandatory step in RIP-Seq and CLIP-Seq data analysis. Reads aligning to multiple genomic positions are usually removed [52]. However, such multi-mapped reads can be used to identify regulatory RNA sites localized within repetitive regions [53]. To filter out PCR duplicates, reads mapping to the same genomic position are collapsed; UMIs, when present, can be used to avoid removing natural read duplicates, common in case of high sequencing depth [54].

In RIP-Seq experiments, target RNAs can be identified either by transcript enrichment analysis of IP versus control samples [55–57], which can be performed using procedures commonly adopted in differential expression analysis, or with ad-hoc peak-calling tools [58,59]. Binding site identification in HITS-CLIP experiments is usually accomplished by means of peak-calling approaches, that identify regions which are enriched in reads with respect to their genomic context (gene, transcript, metagene region) [59], the background represented by control experiments (input, IgG, mock IP) [60], or baseline expression profiles [59]. Single-nucleotide resolution in HITS-CLIP data analysis can be achieved by looking for statistically significant CIMS [59]. Such resolution in binding site identification can also be attained in PAR-CLIP, by searching for transition events that are not likely to be caused by sequencing noise, single nucleotide polymorphisms or contamination [61]. The first nucleotide of reads from iCLIP and eCLIP experiments most often marks the truncation site and it is therefore located one nucleotide after the cross-linking site. Statistically significant CITS can be identified by using tools like iCount [62] and PureCLIP [63].

## *In silico* prediction of protein–RNA interactions

The outcome of the data analysis obtained by means of CLIP-Seq experiments is of enormous value in approaching the study of a potential RBP with unknown RNA partners. However, there are two major drawbacks in limiting the investigation to CLIP-Seq approaches: the protein regions in direct contact with the target RNAs remain unknown, and there are not sufficient data to speculate on the strength of the interaction for each protein–RNA pair. The use of predictive algorithms such as *cat*RAPID would integrate the results of a CLIP-Seq experiment with this information [24] (Figure 1).

### *cat*RAPID

*cat*RAPID is an algorithm able to compute protein–RNA interaction propensities with strong predictive power (area under the Receiver Operating Characteristic curve of 0.78 on >1 000 000 interactions) [64], through the calculation of secondary structures, hydrogen bonding and van der Waals contributions. The algorithm was trained on PDB crystals [24] and was later adapted to predict CLIP-Seq interactions with long non-coding RNAs [65]. For large proteins (>750 amino acids) and RNAs (>1000 nucleotides), the algorithm fragments sequences into overlapping segments and computes the interaction propensity through the analysis of physical–chemical properties and secondary structures of the molecules. According to the chosen implementation (Table 1), the method can either reconstruct the overall interaction propensity score for each protein–RNA pair [66] or rank the fragments according to the predicted interaction strength [67]. The outcome of this analysis allows to map both protein and RNA binding sites and to estimate the overall strength of the interactions [68], overcoming the limitations of CLIP-Seq techniques mentioned above. If used in combination with CLIP-Seq data relative to the protein of interest, *cat*RAPID can be employed to select the best targets based on calculated binding strength, but it could be also useful in predicting putative targets that are not expressed within the cellular system. If no information about the RNA targets is available, *cat*RAPID can represent a promising tool for the investigation of the protein's genome-wide RNA-binding potential against an RNA sequence library. Here we suggest a pipeline that could be followed in such circumstance:

1. If the RNA binding potential of a given protein is unknown, *cat*RAPID *signature* [71] can be used to predict it, along with the putative RNA-binding regions. This approach is particularly recommended if a potential RBP needs to be selected from a panel of candidate proteins;
2. *cat*RAPID *omics* [69] can then be used to predict the interactions between the protein and a precompiled or custom RNA library. The result is a ranked list of protein–RNA pairs;
3. If the protein of interest is human, its co-expression with the putative interactors in different tissues can be evaluated using *cat*RAPID *express* [70];
4. Once the most promising binding partners have been identified, *cat*RAPID *strength* can be employed to evaluate the strength of each interaction [67];
5. Finally, *cat*RAPID *fragments* [66] can be run on the highest scoring protein–RNA pairs to predict the binding sites. Interactions with long RNAs can be analyzed using *Global Score* [65] or *omiXcore* [68].

**Table 1:** A summary of the different *cat*RAPID implementations

Name of the algorithm	Description	Input	Output
*cat*RAPID *fragments* [66]	It divides inputted protein and RNA into fragments and computes the interaction propensity between each fragment.	• A protein sequence in FASTA format. • An RNA sequence in FASTA format.	• Interaction profile plot^a^. • Interaction matrix^b^. • Table of interacting protein–RNA fragments.
*Global Score* [65] *omiXcore* [68]	A variant of *cat*RAPID fragments calibrated on CLIP-Seq data, it is able to predict interaction with >1000 nt long RNAs and to provide an overall interaction score.	• A protein sequence in FASTA format. • An RNA sequence in FASTA format.	• Interaction profile plot^a^ • Interaction matrix^b^. • Table of interacting protein–RNA fragments.
*cat*RAPID *omics* [69]	It computes the interactions between a molecule (protein/RNA) and the reference set (transcriptome/nucleotide-binding proteome) of a model organism.	• Protein/RNA sequence in FASTA format. • Reference set of protein/RNA sequences.	• Graphical representation of protein sequence/domains. • Pie chart with ranking distribution^c^. • Table of interacting protein–RNA pairs.
*cat*RAPID *express* [70]	It allows the identification of co-expressed protein–RNA pairs in human tissues.	• A protein sequence in FASTA format • An RNA sequence in FASTA format. • (Only one protein sequence or one RNA sequence is required for the omics option).	• Correlation coefficient representing the coexpression of the protein–RNA pair. • Interaction heatmap^d^. • Table of tissue expression.
*cat*RAPID *signature* [71]	It scans a protein sequence for RNA-binding regions.	• One or more protein sequences in FASTA format.	• Overall binding score. • Binding propensity plot^e^.
*cat*RAPID *library* [69]	It allows the creation of a new reference set for *cat*RAPID omics.	• One or more protein or RNA sequences.	• A library ID that can be used in catRAPID *omics.*
*cat*RAPID *strength* [67]	It computes the interaction strength of a protein–RNA pair with respect to a reference set of sequences of similar length.	• A protein sequence in FASTA format • A RNA sequence in FASTA format.	• Table of interaction strength (significance of interaction propensity). • Cumulative distribution function plots of protein-RNA interaction score^f^.

a The interaction profile plot represents the interaction score (*y*-axis) of the protein along the RNA sequence (*x*-axis), giving information about the transcript regions that are most likely to be bound by the protein;

b The interaction matrix is an heatmap showing the interaction propensity between each possible fragment of the protein (*y*-axis) and the RNA (*x*-axis);

c The pie chart shows the proportion of targets having High, Moderate and Low star rating score. Star rating score weights the interaction based on the interaction propensity, the presence of RNA/DNA binding domains and the presence of known RNA motifs;

d The interaction heatmap shows the interaction score of the individual amino acid-nucleotide pairs;

e The binding propensity plot reports, for each amino acid (*x*-axis), the propensity to be part of a binding region;

f The Cumulative distribution function plots report the interaction score of the query protein–RNA pair within the distribution of the interaction scores from the reference set.

A more detailed explanation of the different algorithms is available on *cat*RAPID tutorial page (http://s.tartaglialab.com/static_files/shared/tutorial.html) and documentation page (http://s.tartaglialab.com/static_files/shared/documentation.html).

By narrowing down the number of potential targets and by suggesting the most likely binding sites, such approach can be employed to guide further experimental and computational analyses. Being a predictive tool, there is always the chance that *cat*RAPID may fail in identifying valid RNA targets. Prediction accuracy depends on the set provided to train the algorithm. As more and more data become available, retraining of the algorithm will be necessary to achieve better performances.

### *cat*RAPID alternatives

*cat*RAPID is only one of several possible computational methods developed for predicting protein–RNA interactions. We would like to mention here some valid alternatives:

- LncPro [72]: similar to *cat*RAPID in the employment of RNA secondary structure, hydrogen-bonding and van der Waals interaction propensities. It is designed to predict whether a specific long non-coding RNA interacts with one or more protein sequences. Propensities are calculated for both protein and RNA and a probability score ranging from 0 to 100 is generated;
- RPISeq [73]: protein and RNA sequences are encoded into features that are then used to train Support Vector Machine (SVM) and Random Forests classifiers;
- RPI-Pred [74]: it combines RNA and protein sequences with predicted or actual 3D structures. The features are then used to train an SVM classifier;
- iDeepS [75]: a deep learning-based method that exploits convolutional neural networks (CNNs) trained on RNA sequences and predicted secondary structures. At the end of the pipeline, a classification layer is responsible for RBP binding sites prediction. Deep learning models are generated individually for each RBP based on available CLIP-Seq data, allowing the formulation of predictions on a limited set of proteins.

## Functional characterization of RNA targets and binding sites

Once the RNA targets of a protein have been determined, further analyses are necessary in order to verify the reliability of the results and to gain insights into the biological function of the RBP. Both tasks can be approached by looking at the function of target RNAs. A common way to do that is to perform an Over Representation Analysis (ORA) [76], which consists of identifying over-represented functional categories in a list of genes. Enrichment is evaluated against a background composed of all the expressed genes. A more sophisticated approach, called Gene Set Enrichment Analysis (GSEA) [76], involves ranking genes based on a certain score and evaluating if some categories are enriched at the top or the bottom of the ranked list. An implementation of this method that is specific for CLIP-Seq data is provided by the Seten tool [77]. This program requires as input a set of CLIP-Seq peaks, each with a score assigned by the peak-caller, but it could also work starting from predicted binding sites, as long as an interaction score is provided.

Another common analysis consists in scanning the identified binding sites in order to detect common patterns highlighting RBP binding preferences (motif analysis). Sequence motifs recurring in large sets of binding sites can be discovered using different tools, like MEME-ChIP [78] and SeAMotE [79]. Both tools start from a set of sub-sequences identified in the positive sequence set and evaluate their enrichment with respect to a control sequence set (unbound RNAs). A more recent tool, named mCross, exploits the single-nucleotide resolution offered by CLIP-Seq techniques to enhance the accuracy of *de novo* motif discovery [80].

Sequence alone may not be sufficient to fully explain the binding specificity of an RBP: a sequence motif could be accessible only when put in a proper secondary structure context. Tools like GraphProt [81], ssHMM [82] and BEAM [83] are able to detect motifs encoding both sequence and secondary structure information.

## *In vitro* validation of predicted RBP–RNA interactions

To validate the prediction accuracy of the computational analysis proposed so far, it is ideal to evaluate each interacting pair within a controlled environment. A most accurate validation should start from the screening of potential binders, followed by the precise determination of binding sites and kinetic and thermodynamic parameters, and completed with structural insights into the drivers of the interaction (Figure 1). A comprehensive review of the methodology is beyond the scope of this article and for more details we refer to a number of recent reviews of the field [16,17,84–86].

### Kinetics and thermodynamics of RBP–RNA interactions

As for other molecular interactions, the binding between a protein and an RNA molecule is kinetically characterised by the rate at which they associate (*k*_on_) and dissociate (*k*_off_). Conventionally, the dissociation constant (*K*_d_), which is the ratio between *k*_off_ and *k*_on_ at the chemical equilibrium, is used to express the binding affinity: the lower the *K*_d_, the greater the affinity. It is however important to note that binding pairs with the same *K*_d_ may have different *k*_on_ and *k*_off_ and therefore different binding mechanisms. To add another layer of complexity, many RBPs have multiple binding regions that may vastly differ in their affinity towards the same RNA [87].

There are several established methods for determining the kinetic and thermodynamic parameters of binding (Table 2). Techniques such as electrophoretic mobility shift assay (EMSA) and filter binding assay can be useful to estimate binding affinities with basic molecular biology tools [88,89]. Both these methods represent a viable first step analysis, especially because of short protocols and limited amounts of samples required. The latter criterion can be crucial in protein–RNA interaction studies, since some RBPs can be very difficult to isolate, tagged RNA synthesis can be expensive and advanced methods for more reliable and accurate determination of molecular binding characteristics generally have specific sample requirements and higher operation costs. Despite these advantages, since EMSA and filter binding experiments are performed within conditions very distinct from the *in vivo* ones (polyacrylamide gel and nitrocellulose filter, respectively), more reliable kinetic data may be obtained by other techniques. Examples of such methods are bio-layer interferometry (BLI), multi-channel surface plasmon resonance (SPR), microscale thermophoresis (MST), the employment of fluorescence resonance energy transfer (FRET), and the most recent switchSENSE (Table 2, [86–89, 95]). Some of these require the labelling of one of the interactors with fluorescent dyes, as in the case of MST and FRET, or sample immobilisation on biosensors, as needed for SPR and BLI experiments. These requisites can impose certain structural restraints and thus compromise the binding and the results obtained. Comparing how the same RBP binds to RNA molecules that differ only in one nucleotide can help the identification of the RNA portion physically interacting with the protein. However, the determination of the exact nucleotides involved in the binding can be highly challenging without structural studies. Once the RNA targets have been precisely characterised, selective mutations on the protein region thought to be responsible for the binding may be useful to determine which amino acids are directly responsible for the interaction. The same approach could be extended to mutating selected nucleotides or regions of the RNA molecules for further characterisation of the binding spots without obtaining an atomic resolution structure.

**Table 2:** Methods for *in vitro* characterisation of protein–RNA binding

Method	Principle of detection	Sample requirements	Detection range	Sample capacity	Direct measurements
*EMSA* [90]	Detection of RNA–protein complex’ electrophoretic mobility properties, typically different compared to free RNA.	• Labelled RNA. • nM concentrations of RNA and protein.	≥10^−18^ mol RNA.	0.5–500 µl depending on electrophoresis setup.	*K*_d_, *n*
*Filter binding assay* [88]	Quantification of ^32^P-labelled RNA via imagine screen or scintillation counter.	• About 0.1 µM labelled RNA (usually with ^32^P). • Purified protein serial dilutions.	≥10^−15^ mol RNA.	Multi-well plate dot-blot setup.	*k*_on_, *k*_off_, *K*_d_, *n*
*Fluorescence anisotropy* [91,92]	Changes in fluorescence anisotropy or polarisation of excitation light upon binding.	• Fluorescent labelling of one of the partners. • 1 nM RNA.	nM ranges of fluorophores.	Multi-well plates.	*K*_d_
*FRET* [93,94]	Energy transfer of between fluorophores detected as a change in fluorescence intensity.	• Two fluorophores, either one on each partner or strategically placed on one for structural studies.	single-molecule experiments.	Single molecule to multi-well plates.	*K*_d_, *k*_on_, *k*_off_, distance between fluorophores.
*SPR* [95]	Variations in the refractive index of polarised laser light upon molecular binding.	• About 200 µl 25 nM RNA/sensor. • Variable conc. of protein (ideally 100-times *K*_d_), up to 4 ml of sample. • Immobilisation of one partner required.	1 pM < *K*_d_ < 1 mM	Up to 16 channels with microfluidics.	*K*_d_, *k*_on_, *k*_off_
*BLI* [96]	Detection of the variation of refracted white light upon the binding of the interaction partner to the immobilised ligand on the optical fibres.	• 1–50 µg/ml of ligand, immobilised on biosensor. • 1 nM–µM of receptor. • 5–250 µl of sample per measurement.	1 nM < *K*_d_ < 10 mM	Single channel, 5 min per measurement (BLItz) or multi-well plate, 1–8 simultaneous channels.	*K*_d_, *k*_on_, *k*_off_
*MST* [97]	Variations in temperature-induced fluorescence emission of a target as a function of the concentration of a non-fluorescent ligand.	• 1–20 µl, nM–µM concentrations. • Fluorescent labelling.	pM < *K*_d _< mM	Up to 96 samples per run in a multi-capillary system.	*K*_d_
*switchSENSE* [98]	Voltage-dependent variations of the movement of short fluorescent DNA nanolevers attached to a gold surface upon binding of an analyte.	• Immobilisation of one binding partner. • 20 µl of 1 µM RNA for biochip saturation. • 250 µl of 0.2 µM protein.	nM < *K*_d_ < µM	Four flow channels with six microelectrodes for sampling per chip	*K*_d_, *k*_on_, *k*_off_, *R*_h_
*ITC* [99]	Measuring the heat consumed/released during titration of sample with the ligand in regard to reference cell.	• 200 µl–2 ml of 1-2 µM receptor. • 40–500 µl 10× concentration ligand.	• *K*_d_ > nM (direct measurements). • *K*_d_ > pM (competitive binding).	single cell.	*K*_d_, Δ*H*, *n*

Kinetic constants are measured directly and are used as basis for equilibrium thermodynamic parameters calculations, apart from ITC where the reaction enthalpy can be obtained without relying on kinetic data. *K*_d_: equilibrium dissociation constant; *k*_on_: association rate constant; *k*_off_: dissociation rate constant; *n*: stechiometry of binding; *R*_h_: hydrodynamic radius (radius of a theoretical sphere with the same translational diffusion coefficient); Δ*H*: reaction enthalpy.

Kinetic studies are essential for determining the binding propensity and validating different binding partners [10]. However, experiments conducted *in vitro* with isolated components often do not allow studies under physiologically relevant conditions. Isolated proteins can be sensitive to higher temperatures, especially under prolonged experiments that far exceed their in-cell lifespans. Since chemical equilibriums are temperature-dependent, *in vivo* kinetics may thus significantly differ from those measured *in vitro*. The thermodynamics of binding can also reveal the energetic landscape of protein–RNA interactions [100–102]. It is therefore very important to measure the thermodynamics parameters directly, when experimentally possible, or to calculate them. Isothermal titration calorimetry (ITC) allows direct measurements of the equilibrium constant, stoichiometry and reaction enthalpy (ΔH) at a given temperature [99]. Using the van't Hoff equation, these can then be used to determine the temperature dependency of the equilibrium constant. SPR and MST also allow calculation of thermodynamic data based on the stable temperature of the measurement cell, while BLI is considered less reliable.

### Structural approaches of studying RBP–RNA interactions

Kinetic and thermodynamic data obtained from interaction studies provide a good numerical description of the binding and, through the use of RNA-centric methods, a library of RNA sequences with high binding propensities [103]. However, protein and RNA sequences alone may not be sufficient to fully characterise their binding specificity. This is especially true in the case of non-canonical RBPs that do not contain a consensus RNA-recognition sequence [104]. RNA structure has been shown to be the driver behind most non-canonical RBP binding events, with highly structured RNAs having bigger protein interactomes [105,106]. The approaches to determine the structure of separate components have been extensively reviewed [107,108]. Among the most pertinent techniques to determine the macromolecular structure of a complex, there are X-ray crystallography, nuclear magnetic resonance (NMR) and cryogenic electron microscopy (cryo-EM) (Table 3). However, the definition of structural details by these methodologies can be challenging [107]. A large number of RBPs contain intrinsically disordered regions and tend to form macromolecular condensates, making their crystallisation impractical; NMR, that bypasses the need for crystals, is dependent on the molecular weight of the complexes that can make relaxation times slow and signal-to-noise ratio poor; obtaining results at atomic resolution with cryo-EM remains difficult [108]. These limitations make a strong case for the employment of complementary structural biology techniques [107]. A particularly promising development is the advance of small angle scattering and computational methods for data analysis, in particular small angle neutron scattering (SANS) [109]. Selective deuteration enables a higher degree of contrast between binding partners and can therefore provide a rough position for each component of the complex [110]. Data obtained from the above mentioned methods can be used as structural restraints for molecular dynamics simulations and data-driven docking [111,112] and can be integrated together in a hybrid multi-level approach for studying RNA-protein complexes, thus completing the full circle of integrated methodologies [75,113,119,120].

**Table 3:** A short overview of the major structural biology techniques with a comparison of their advantages and disadvantages for the study of protein–RNA interactions

Method	Principle of detection	Resolution	Sample requirements	Pros/Cons
*NMR* [111,114]	Detection of the electric current, induced by the magnetization of the non-equilibrium spins in a magnetic field. Upon Fourier transform, the results can be used to determine structural constraints and produce a molecular model.	atomic (<2 Å).	• Isotope labelling, side-chain deuteration essential for larger complexes to avoid lengthy relaxation times. • Protein concentration varies according to MW.	• Solution-based, can observe time-resolved experiments and kinetics, most accessible on the list, possibilities of differential isotope labelling, saturation transfer experiments and more. • Poor signal-to-noise ratio, line broadening and complex spectra with higher molecular mass complexes.
*X-ray crystallography* [115,116]	Detection of diffracted X-ray photons, scattered by the crystal, from which an 3D electron density map is calculated, which is then used to build the molecular structure model.	atomic (<2 Å).	Crystals of the protein–RNA complex, frozen in liquid nitrogen.	• Highest resolution limit with free electron lasers. • Relies on quality crystals, often difficult to obtain.
*Cryogenic electron microscopy* [117]	Based on electron microscopy, the sample images are grouped into specific projections, with a 3D model calculated based on them.	high (<5 Å).	Monodisperse sample blotted onto grids and frozen under cryogenic conditions.	• Solution based, flexible buffer components, no need for crystals etc. • Maximum resolution limit around 3.5 Å for molecules with a MW ∼50 kDa. • Very difficult to obtain sufficient quality data for determination of structures of elements with MW < 150 kDa under the resolution of 5 Å.
*Small angle scattering* [110,112,118]	Detection of diffracted X-ray photons (SAXS) or neutrons (SANS) on sample solutions under small angles (typically <10°), from which a scattering curve and a 3D shape can be calculated.	medium (>10 Å).	• Monodisperse sample, dilution series from 1 mg/ml to 20 mg/ml. • Possible deuteration or isotope labelling for SANS studies.	• Investigation of molecule shape as well as other information, selective deuteration can provide valuable contrast (SANS). • Need for monodisperse sample, rather high protein concentrations for the dilution curve, no exact molecular structure.

## Perspectives

- Our understanding of many physiological and pathological phenomena cannot be exempted from an in-depth knowledge of protein–RNA interactions underlying them.
- Such comprehension, which goes from the identification of targets RNAs to binding modes characterization, requires a multidisciplinary approach involving biochemistry, molecular biology, bioinformatics and physics.
- As new and more powerful high-throughput methods and predictors are being developed, an integrated and productive usage of both approaches becomes more and more feasible. For instance, predictive tools such as *cat*RAPID, which is general enough to be applied to any protein–RNA pair, could also be employed to improve the specificity of omics studies.

## Abbreviations

BLI: biolayer interferometry
CIMS: crosslinking-induced mutation sites
CITS: crosslinking-induced truncation sites
CLIP: cross-linking followed by immunoprecipitation
CLIP-Seq: CLIP followed by followed by high-throughput sequencing
eCLIP: enhanced CLIP
FRET: Förster resonance energy transfer
HITS-CLIP: high-throughput sequencing CLIP
iCLIP: individual nucleotide-resolution CLIP
IP: immunoprecipitation
MST: microscale thermophoresis
NMR: nuclear magnetic resonance
PAR-CLIP: photoactivatable ribonucleoside CLIP
RBP: RNA-binding protein
RIP: RNA immunoprecipitation
RIP-Seq: RIP followed by high-throughput sequencing
SPR: surface plasmon resonance
SVM: support vector machine
UMI: unique molecule identifier
